# Expert perspectives on the requirements for implementing prehospital peripheral nerve blocks: a survey among members of the European Society of Regional Anaesthesia (ESRA)

**DOI:** 10.1186/s12871-026-04093-3

**Published:** 2026-07-15

**Authors:** Christine Gaik, Alan JR Macfarlane, Eleni Moka, Axel R Sauter, Ann-Kristin Schubert, Thomas Volk, Hinnerk Wulf, Benjamin Vojnar

**Affiliations:** 1 Marburg University, Marburg, Germany; 2 Department of Anesthesiology and Intensive Care Medicine, University Hospital Giessen and Marburg, Campus Marburg, Baldingerstraße, Marburg, 35033 Germany; 3 Department of Anesthesia, Glasgow Royal Infirmary, Glasgow, UK; 4 School of Medicine, Dentistry and Nursing, University of Glasgow, Glasgow, UK; 5 Department of Anesthesiology, Creta Interclinic Hospital, Hellenic Healthcare Group (HHG), Heraklion, Greece; 6 Department of Anesthesia and Intensive Care Medicine, Oslo University Hospital, Oslo, Norway; 7 Department of Anesthesiology and Pain Medicine, Bern University Hospital, Inselspital, University of Bern, Bern, Switzerland; 8 Department of Anesthesiology, Intensive Care and Pain Therapy, Saarland University Medical Center and Saarland University Faculty of Medicine, Homburg, Germany

**Keywords:** Prehospital nerve block, Pain management, Prehospital regional anesthesia, Trauma analgesia, Point-of-care ultrasound

## Abstract

**Background:**

Evidence on prehospital regional anesthesia (RA) is limited, with only a few trials comparing peripheral nerve blocks (PNBs) to standard opioid analgesia. Although well established in hospitals, their use in prehospital care remains rare. This survey aimed to capture expert perspectives on prerequisites, barriers, and preferred block techniques for broader implementation of prehospital PNBs.

**Methods:**

Members of the European Society of Regional Anaesthesia (ESRA) were invited to participate in an anonymous online survey. The 30-item questionnaire collected demographic data, professional background, information on the organization of emergency medical services (EMS), and ultrasound availability. Participants were also asked about their experience with in-hospital and prehospital RA, perceived training requirements, and necessary equipment. Finally, respondents assessed the advantages, disadvantages, and suitability of specific RA techniques for prehospital scenarios. Data were analyzed descriptively and are presented as absolute numbers and percentages.

**Results:**

A total of 415 fully completed questionnaires were included in the analysis. 82% (342/415) of respondents supported prehospital RA, but only 20% (73/415) reported its current use in their EMS area. Onethird of this group had personally performed RA procedures, with fascia iliaca compartment blocks (FICB) and femoral nerve blocks (FNB) being the most frequently used techniques. The most commonly cited key requirements for performing prehospital RA included ultrasound devices (87%; 359/415) and lipid solution (68%; 282/415). 58% (240/415) of respondents reported ultrasound availability in prehospital care. 63% (262/415) believed only specialists or residents with additional training should perform PNBs.

**Conclusion:**

While most respondents supported the concept of prehospital RA, only few indicated that such approaches are currently implemented in their local EMS systems. FICB and FNB were identified as the preferred options for managing proximal femur fractures. Key concerns regarding implementation included limited team experience, time constraints, and procedural complications such as local anesthetic systemic toxicity (LAST), emphasizing the need for structured training, clear protocols, and expert guidance to ensure safe implementation.

## Introduction

Within anesthesiology, regional anesthesia (RA) is a well-established and widely used technique in perioperative patient care. Peripheral nerve blocks (PNBs) are utilized both as a stand-alone procedure and as adjunctive analgesia in combination with general anesthesia (GA), providing significant benefits in both preoperative and postoperative settings [1]. In the context of emergency trauma care, peripheral nerve blocks can also be performed for analgesia in the emergency department (ED) [2]. 

However, unlike other invasive procedures in prehospital emergency care – such as peripheral venous cannulation, endotracheal intubation, intraosseous access, or needle decompression – PNBs, which are arguably less invasive, are rarely performed in prehospital settings. Their application is generally confined to isolated cases or research studies. Evidence regarding the prehospital use of RA techniques is limited [3–5]. Additionally, relatively few studies exist that compare PNBs with other analgesic modalities – such as opioids, ketamine, or inhaled analgesics – in out-of-hospital settings.

Therefore, this international survey aimed to capture expert opinions and clinical experience regarding the potential implementation of PNBs in prehospital emergency care. Since PNBs are primarily performed by anesthesiologists in hospital settings across most European countries and are not part of standard emergency medicine training, members of the European Society of Regional Anaesthesia (ESRA) were considered an appropriate expert group for this exploratory study.

Based on respondent input, the survey sought to identify key prerequisites, perceived barriers, and potential strategies for integrating PNBs into prehospital practice. Given the current absence of national recommendations for prehospital RA, the findings of this study may help define essential requirements and support the development of future clinical guidance. To the best of our knowledge, no comparable survey has been conducted to date.

## Methods

### Ethics approval and setting

This cross-sectional study was conducted among members of the ESRA from June 2024 to January 2025. The study was prospectively registered in the German Clinical Trials Register (DRKS) under the registration number DRKS00034436. Registration was completed on 24 June 2024, and the first participant responded to the survey on 29 June 2024. Ethical approval was obtained in advance from the Ethics Committee of the Medical Faculty, Philipps University of Marburg (Reference: 24/63 ANZ; approval granted on 25 March 2024). This manuscript adheres to the current Strengthening the Reporting of Observational Studies in Epidemiology (STROBE) guidelines.

### Participants

Members of ESRA were invited to participate in an anonymous, web-based survey. Participation was entirely voluntary, and no financial or other incentives were provided. The survey invitation was distributed via a general ESRA newsletter on 29 June 2024, with reminder emails sent on 19 August 2024 and again on 22 January 2025, shortly before ESRA World Day 2025.

### Study design

The survey was developed using a secure, web-based platform (SurveyMonkey, San Mateo, California, USA) and consisted of 30 questions, some of which incorporated conditional logic. In specific cases, subsequent questions were displayed only if the previous response met predefined criteria. Participants accessed the survey via a link that directed them to the study’s homepage on the SurveyMonkey platform. The survey began with a brief introduction outlining the purpose and objectives of the study, followed by a consent declaration that participants could accept or decline by selecting the appropriate option. To ensure data integrity, web cookies were used to restrict responses to one per participant. The order of the questions was identical for all respondents.

### Aim of the study

The study aimed to evaluate expert opinions on the feasibility and key requirements for implementing PNBs in prehospital emergency care. This included identifying potential barriers, safety considerations, and training needs associated with prehospital RA, as well as determining which block techniques are regarded as most suitable for common indications in this setting. In addition, the survey examined expert perspectives on the relevance and availability of ultrasound equipment and on the qualifications and scope of practice required to safely perform PNBs outside the hospital environment.

### Data management and analysis

Data were managed and analyzed using Microsoft Excel 2013 (Microsoft Corporation, Redmond, WA, USA). Only fully completed questionnaires were included in the analysis. To proceed through the questionnaire, participants were required to answer all preceding items. As this study was designed as an exploratory, opt-in survey aimed at capturing expert perspectives rather than estimating population parameters, no formal sample size calculation was performed. In accordance with recommendations from the American Association for Public Opinion Research (AAPOR) for non-probability online surveys, traditional margin-of-error estimates and sample size calculations are generally not applicable to this type of survey design. Instead, the focus was placed on transparent reporting of recruitment procedures, response numbers, and descriptive analysis of the collected data, as the study was designed for descriptive analysis only and no inferential statistical analyses were planned.

## Results

The initial survey invitation was emailed to 26,022 ESRA members and achieved an open rate of 45%. Following a reminder email sent to 26,067 members, the open rate increased slightly to 46%. In total, 651 individuals accessed the survey link. Among these, 550 submitted responses, yielding a response rate of 85% among those who accessed the survey. After exclusion of 135 incomplete submissions, 415 fully completed questionnaires were included in the final analysis. This corresponds to an overall response rate of 3% based on the total mailing list. Respondents’ demographics, professional roles, current workplaces, and experience in anesthesia are summarized in Table 1.

**Table 1:** Data on respondents’ demographics, job positions, current workplaces, and experience as anesthetists

	*N*	%
Country		
Germany	59	14%
United Kingdom (UK)	32	8%
Portugal	20	5%
Greece	17	4%
Italy	16	4%
Belgium	15	4%
India	14	3%
Pakistan	13	3%
Spain	12	3%
Switzerland	12	3%
Mexico	11	3%
France	10	2%
Others*	196	47%
Job position		
Physician	394	95%
Nurse	4	1%
Paramedic	2	0%
Other position	15	4%
Workplace / Hospital of the respondents		
Hospital with more than 900 beds and/or university hospital	148	36%
Hospital with 300–900 beds	131	32%
Hospital with fewer than 300 beds	106	26%
Other workplaces	28	7%
Experience as an anesthetist		
Less than 5 years	50	12%
5 to 10 years	105	25%
11 to 20 years	127	31%
For more than 20 years	122	30%
I am not an anesthetist	9	2%

*Only countries with at least 10 respondents are listed in this table

### Prehospital work in emergency medicine

When asked about the primary providers of prehospital emergency care in their country, 16% (66/415) of respondents indicated that only non-physician personnel (e.g., paramedics, nurses) deliver care. In contrast, 12% (50/415) reported that prehospital emergency care is provided exclusively by physicians. The majority – 66% (275/415) – stated that care is delivered by a combination of physicians and non-physician providers. Additionally, 6% (24/415) were unsure and selected ‘I don’t know’.

### Availability of ultrasound in EMS

In response to the question regarding the general availability of ultrasound devices in their immediate ambulance service area at the prehospital stage, 34% (142/415) reported ‘sporadic’ availability, 17% (71/415) indicated ‘frequent’ availability, and 7% (27/415) noted ‘nationwide/area-wide availability’. Meanwhile, 31% (128/415) stated that no ultrasound equipment is available prehospitally, and 11% (47/415) were unable to provide precise information, selecting ‘I don’t know’.

### In-hospital experience with peripheral regional anesthesia

**Fig. 1:**
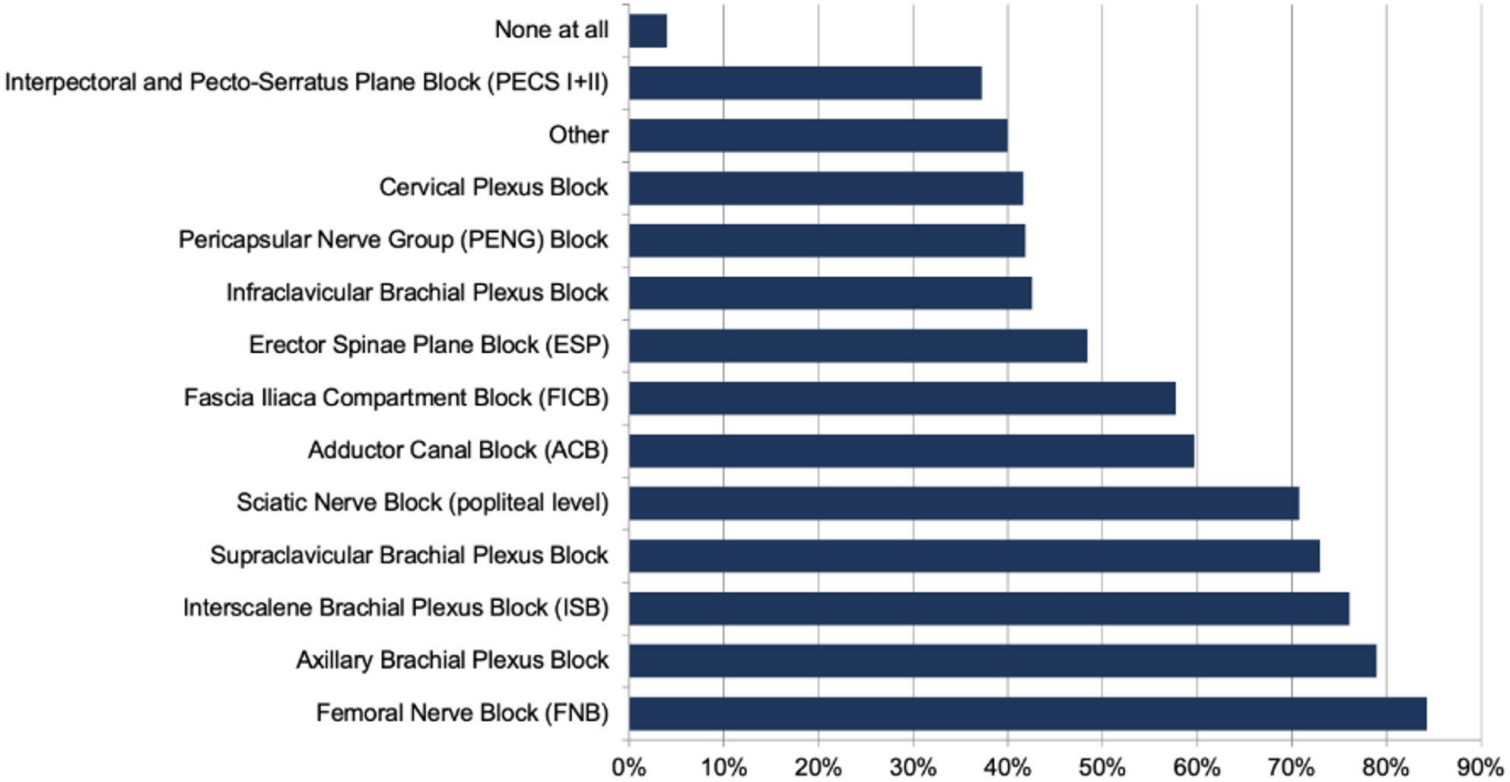
Overview of PNB performed independently by respondents in clinical practice. Multiple responses possible (415 responses)

Regarding respondents’ individual experience with peripheral RA in the hospital setting, 67% (277/415) described their level of expertise as ‘advanced (supervising others)’. Another 29% (122/415) reported having ‘basic skills (need help with advanced blocks)’, while 4% (16/415) indicated having ‘no experience’ with peripheral RA. We inquired about PNBs currently performed independently and without supervision in the hospital setting. The distribution of reported nerve block techniques is presented in Fig. 1.

A total of 59% (243/415) of respondents reported using ‘anatomical landmarks’ with ultrasound as their primary method for needle guidance in peripheral RA procedures. ‘Dual guidance with ultrasound and a nerve stimulator’ was indicated by 33% (137/415). Another 4% (18/415) relied only on ‘anatomical landmarks without ultrasound or a nerve stimulator’, while 2% (10/415) used ‘anatomical landmarks with a nerve stimulator’. Additionally, 2% (7/415) stated that they had ‘no experience’ in this area.

### Prehospital experience with peripheral regional anesthesia

**Fig. 2:**
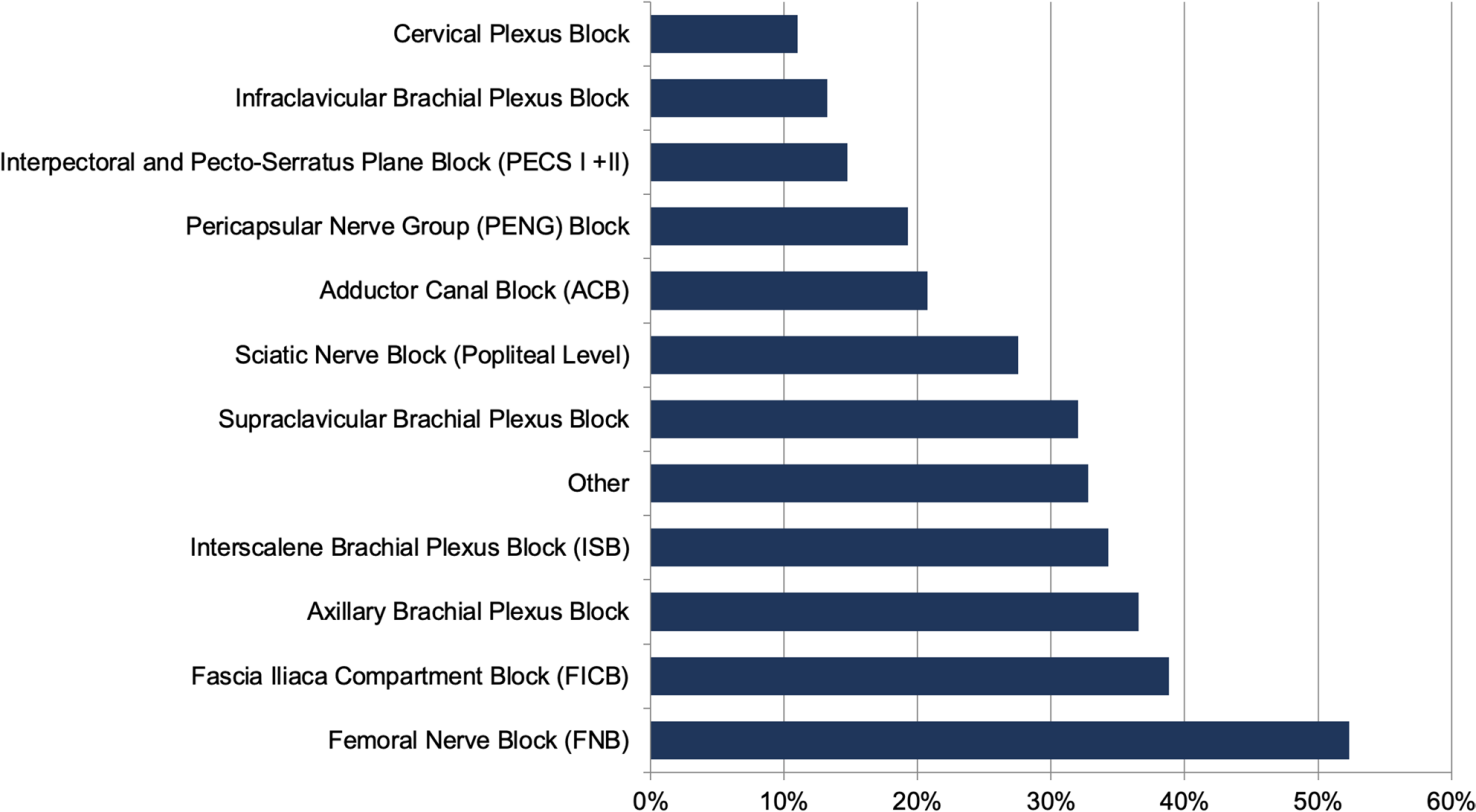
Overview of prehospital PNB procedures performed, showing wide range. Multiple responses possible (133 responses)

Four fifths (82%, 342/415) of respondents stated that prehospital RA is not currently performed in their region, while 18% (73/415) reported that such procedures are already being conducted in their area. Participants were also asked to rate their individual experience with prehospital PNBs. A total of 68% (282/415) reported having no experience in this area. Meanwhile, 20% (81/415) described their experience as ‘advanced’ (supervising others), and 13% (52/415) indicated that they possess ‘basic skills’ (requiring assistance with advanced blocks). Among respondents reporting ‘advanced’ experience in prehospital PNBs, 32% (26/81) indicated that they were currently working as prehospital emergency physicians. Physicians who reported ‘advanced’ experience or ‘basic skills’ in prehospital PNBs were further asked about the specific types of PNBs they perform in prehospital settings (Fig. 2).

### Perspectives on the future use of prehospital regional anesthesia

Overall, 82% (341/415) of respondents regarded the prehospital application of PNB procedures as beneficial in principle, whereas 18% (74/415) did not share this view.

Participants were also asked which type of physicians they believe should be permitted to perform prehospital peripheral RA. Nearly two-thirds (63%; 262/415) indicated that only ‘residents and/or specialists (with extra training)’ should be allowed to perform prehospital PNBs.

In terms of physician qualifications, 47% (196/415) of respondents stated that only anesthesiologists (residents or specialists) should be qualified to perform prehospital RA, while 53% (219/415) believed that physicians from other specialties should also be eligible to perform PNBs.

When considering non-physician healthcare professionals, 55% (230/415) of participants stated that paramedics, nurses, or other non-physician staff should not be permitted to perform PNBs in the prehospital setting. Meanwhile, 27% (112/415) supported the implementation of prehospital PNBs by non-physician staff ‘only under medical supervision (on-site or via telemedicine)’, and 18% (73/415) believed that non-physician staff should be allowed to perform PNBs ‘independently, after theoretical and practical training’.

**Fig. 3:**
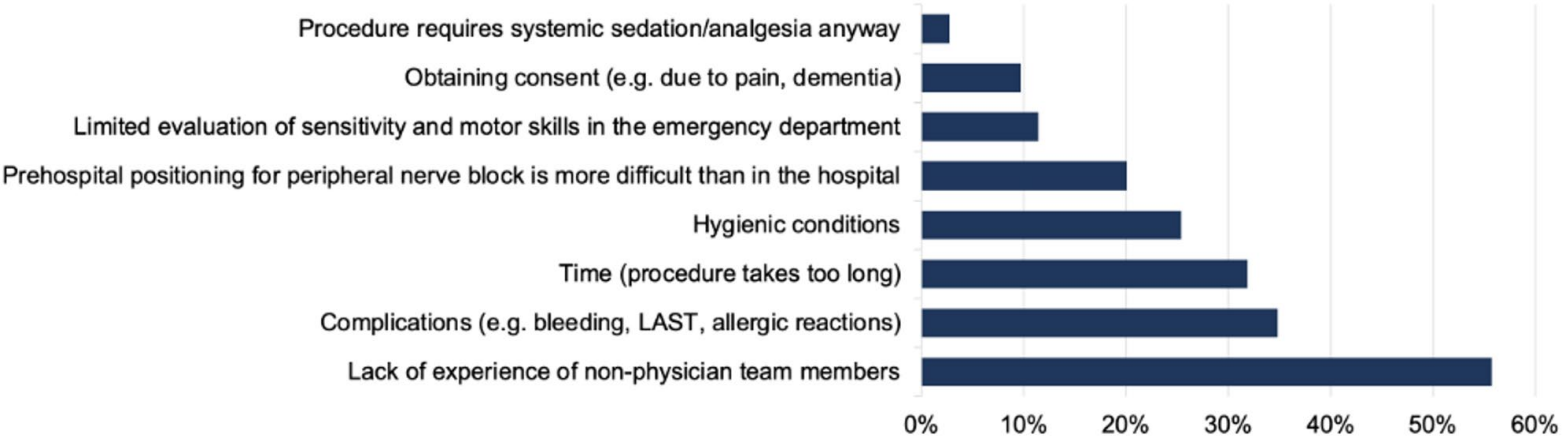
Arguments against performing prehospital peripheral RA. Respondents were allowed to select up to two options (415 responses). LAST = Local anesthetic systemic toxicity

**Fig. 4:**
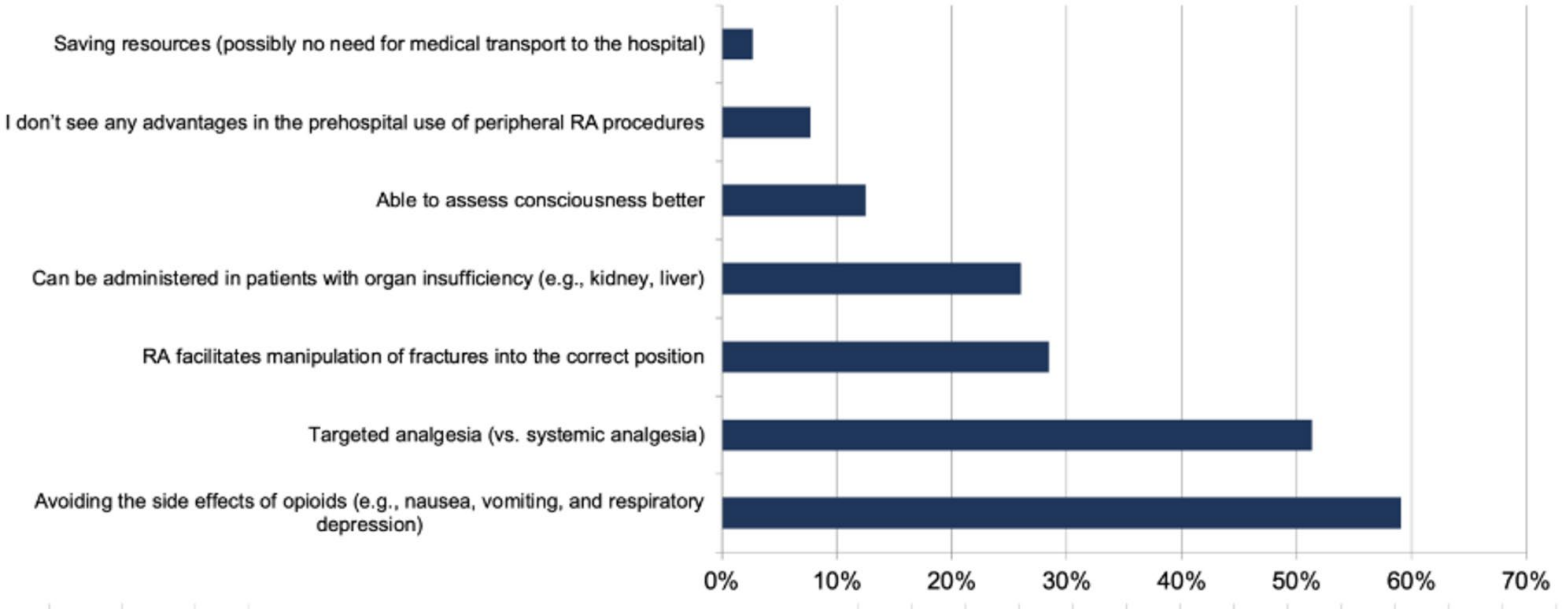
Benefits of prehospital peripheral RA. Two options per respondent were possible (415 responses)

**Fig. 5:**
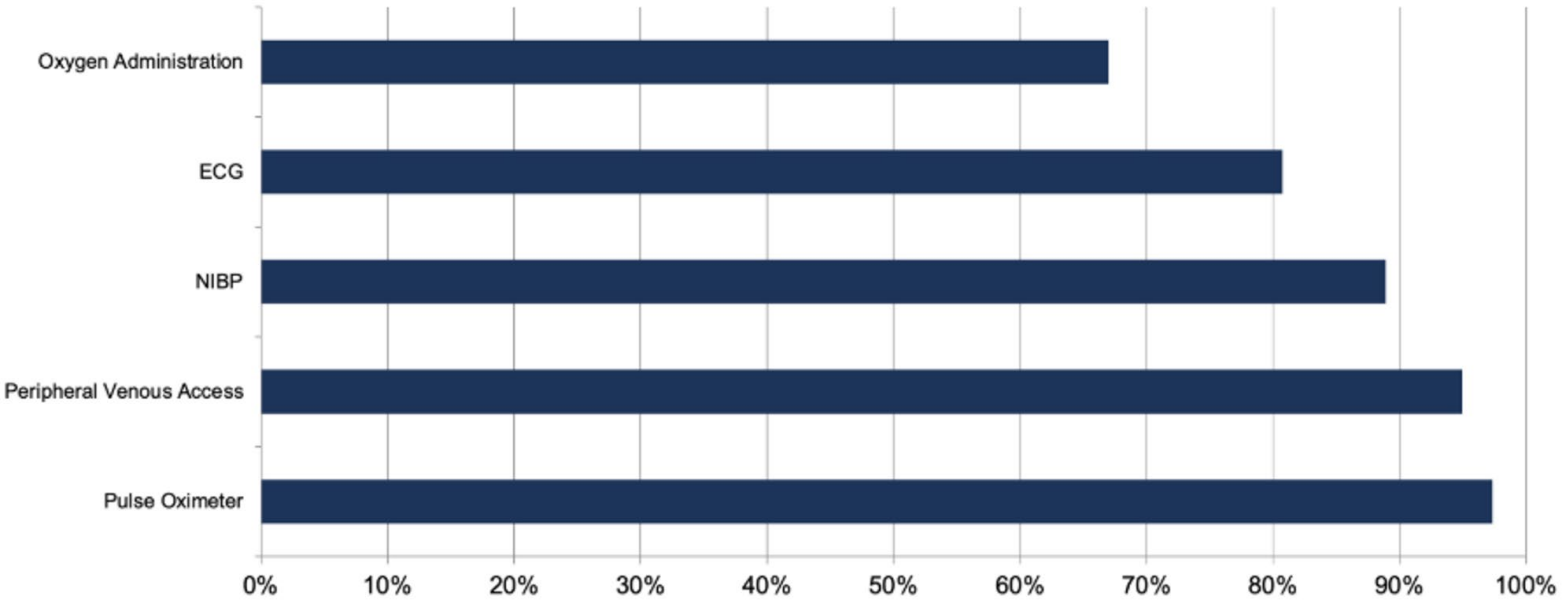
Mandatory requirements for performing prehospital peripheral RA procedures. Multiple responses possible (415 responses). ECG = Electrocardiogram, NIBP = Non-invasive blood pressure measurement

Participants were also asked to share their views on the potential benefits of prehospital peripheral RA, key concerns regarding its implementation, and essential requirements (such as electrocardiogram (ECG) monitoring and non-invasive blood pressure (NIBP) measurement). The results are summarized in Figs. 3, 4, and 5.

Respondents also identified several essential measures required for the prehospital implementation of PNBs. The most frequently mentioned were a pulse oximeter (97%, 404/415), peripheral venous access (95%, 394/415), NIBP measurement (89%, 369/415), an ECG (81%, 335/415), and oxygen administration (67%, 278/415). In addition, 77% (318/415) of respondents reported that travel time to the hospital influences their decision to perform prehospital RA, whereas 23% (97/415) stated that transport time does not affect this decision.

### Options for prehospital peripheral nerve blocks: three clinical case scenarios

In the case of a proximal femoral fracture with normal vital signs, 167 of 415 respondents (40%) indicated they would perform a fascia iliaca compartment block (FICB) in the prehospital setting. Other commonly preferred techniques were the femoral nerve block (FNB), chosen by 79 respondents (19%); the pericapsular nerve group (PENG) block, selected by 66 respondents (16%); and the combination of an FNB with a lateral femoral cutaneous nerve block, reported by 62 respondents (15%). A small group of 35 respondents (8%) stated they would not perform a PNB in this scenario, and 6 respondents (1%) indicated other techniques.

For prehospital analgesia in a patient with an isolated shoulder dislocation and stable vital signs, 220 of 415 respondents (53%) preferred the interscalene brachial plexus block (ISB), while 38 respondents (9%) opted for the supraclavicular brachial plexus block. Additionally, 33 respondents (8%) indicated they would perform a cervical plexus block, an infraclavicular brachial plexus block, or another technique. In contrast, 124 respondents (30%) reported that they would not perform a PNB in this scenario.

For prehospital analgesia in a patient with unilateral rib fractures (ribs 4–6) and stable vital signs, 137 of 415 respondents (33%) stated they would perform an erector spinae plane block (ESPB), while 67 respondents (16%) opted for an intercostal nerve block. Another 95 respondents (23%) chose a paravertebral block (PVB), an interpectoral and pecto-serratus plane block (PECS I + II), or another technique. However, 116 respondents (28%) indicated they would not perform a PNB in this scenario.

## Discussion

Respondents identified FICB and FNB as the most suitable techniques for lower extremity analgesia in potential prehospital applications. Ultrasound was regarded as the most essential prerequisite for performing PNBs, although access to portable devices was reported to be variable. A clear majority emphasized the need for dedicated training prior to any implementation of prehospital RA, while more than half opposed extending such procedures to non-physician personnel. Frequently mentioned concerns included limited team experience, potential delays due to prolonged on-scene times, and the risk of procedural complications such as local anesthetic systemic toxicity (LAST).

With hip fractures in geriatric patients expected to nearly double by 2050, effective pain management is crucial [6–8]. Respondents considered FICB and FNB as the most suitable options for prehospital RA in proximal femur fractures, aligning with ESRA PROSPECT recommendations for pain management in hip fracture repair [9]. A meta-analysis by Slade et al. found FICB provide superior analgesia over opioids and sedatives alone, with minimal adverse events [4]. However, evidence regarding the feasibility and safety of prehospital FNB remains limited, with Raatiniemi et al. highlighting the need for further research comparing FNB to opioids, ketamine, and inhaled analgesics in the prehospital setting [3]. While only a small proportion of respondents generally opposed RA in this scenario, nearly one-third did not support prehospital RA for (isolated) shoulder dislocations and unilateral rib fractures. Nevertheless, Beals et al. demonstrated emergency physicians can quickly learn ultrasound-guided ISB, providing an effective opioid-free alternative in shoulder dislocations, the most common joint dislocation in EDs [10, 11]. However, reliable data on the widespread adoption of prehospital RA remain limited, as the current literature mainly consists of case reports and studies restricted to specific regions or ED settings [12, 13]. 

Ultrasound availability in prehospital care also remains inconsistent, and no comprehensive global or Europe-wide data exist. In North America, prehospital ultrasound remains uncommon despite recent growth [14, 15]. In Europe, prehospital ultrasound availability ranges from 52% in Germany to 69% in Austria, with increasing use in France since 2011 [16–19]. These findings align with our survey, where nearly half of respondents reported ‘sporadic’ or ‘frequent’ access within their ambulance service area. In addition, some respondents indicated uncertainty regarding ultrasound availability within their local EMS systems, suggesting that variable awareness of local infrastructure may have influenced responses regarding the implementation of prehospital RA.

A clear majority of respondents supported specialized training for physicians before performing prehospital RA. While some favor restricting the procedure to specialized physicians, a significant proportion advocate for including residents with targeted training. Future efforts could focus on establishing standardized training pathways that encourage interdisciplinary involvement while ensuring high standards of patient care through structured education [20]. Although this survey did not explicitly address requirements for standardized training, the results may reflect the approach proposed by Chuan et al. Their international Delphi study aimed to develop a consensus-based curriculum for RA training outside formal fellowships, defining core techniques, learning objectives, minimum procedural experience, and key competencies to improve access and patient safety [21]. Building on this concept, the findings of the present survey may also provide a foundation for future Delphi consensus processes to develop recommendations and implementation frameworks for prehospital RA across different EMS systems. Such efforts could define indications, training requirements, equipment standards, legal and regulatory considerations, and standard operating procedures (SOPs) for the safe implementation of prehospital RA.

The need for clearly defined training requirements also depends on the organization of prehospital EMS systems, which differ considerably across Europe [22]. In some countries, physicians play a central role in emergency care, whereas in others, this role is carried out by specialized non-physician professionals, such as paramedics [23, 24]. Our survey confirms this variation, with nearly two-thirds of respondents indicating that prehospital care in their region is delivered through mixed physician-paramedic models. These structural differences may influence the feasibility of implementing prehospital RA. In physician-staffed EMS systems, physicians, often anesthesiologists, may already have experience with PNBs from hospital practice, facilitating implementation. In contrast, paramedic-based systems may require specific protocols and structured training to ensure safe use of RA. Legal frameworks may also influence whether non-physician providers are permitted to perform nerve blocks in the prehospital setting. Therefore, the findings of this survey may be more applicable to physician-staffed or mixed EMS systems.

More than half of the respondents oppose non-physicians performing PNBs in the prehospital setting, reflecting the ongoing debate surrounding prehospital RA administration amid varying EMS structures worldwide. However, studies suggest that trained paramedics and nurses can successfully administer RA in prehospital or ED settings, though often without the use of ultrasound [25–28]. To ensure early analgesia, Regional Anaesthesia UK (RA-UK) supports infra-inguinal FICB for non-physicians, provided they undergo training and adhere to governance protocols [29]. This approach is currently being further evaluated in the RAPID 2 trial, which investigates the clinical and cost-effectiveness of paramedic-administered FICB for hip fractures in prehospital care [30]. Future advancements in artificial intelligence for anatomical structure recognition may improve ultrasound access, benefiting both physicians and non-physicians [31]. 

The training concept applied in the RAPID 2 trial illustrates how such competencies could be implemented in paramedic-based EMS systems [30]. In this study, paramedics underwent a structured training program consisting of online e-learning modules, classroom sessions led by consultant anesthetists, and supervised in-hospital training. During the hospital-based training phase, paramedics performed FICB under clinical supervision before being allowed to administer the technique in the prehospital setting [30]. This approach demonstrates how nerve blocks such as FICB may be introduced into paramedic-based EMS systems while maintaining appropriate supervision and procedural standards. Similar training approaches have also been reported in other studies of paramedic-administered FICB [26, 32]. 

In addition to differences in provider roles, respondents emphasized that successful implementation of prehospital RA will depend on addressing practical and safety-related challenges, including procedural complications, time constraints, and infection risk.

One key safety concern is LAST, which remains difficult to recognize in prehospital settings due to symptom overlap with other conditions. Treating associated symptoms, such as seizures and hemodynamic instability, is already routine for prehospital staff. Structured training, checklists, and standard operating procedures could improve recognition and management [33]. A checklist, provided by the team leader before prehospital RA, could enhance preparation and protocol adherence [34]. 

Lipid emulsions, standard in EDs for lipid-soluble drug intoxications, are not routinely available in ambulances unless physician-staffed [35]. Given the rarity of LAST, a network-based lipid distribution model, as proposed by Gabrieli et al., may offer a practical solution for prehospital systems. This concept involves coordinated access to lipid therapy through regional collaboration between dispatch centers, ambulances, and nearby hospitals, ensuring timely availability of treatment even when lipid emulsions are not routinely stocked on all vehicles [36]. Recent pharmacovigilance analyses indicate that local anesthetic-associated adverse events are rare and that mortality remains exceptionally low, underscoring the continued importance of education and vigilance in clinical practice [37, 38]. Two prehospital LAST cases following FICB have been reported – one resolved spontaneously, while the other was successfully managed with lipid therapy by paramedics [39, 40]. 

In this context, selecting nerve blocks with a favorable safety profile appears important for early implementation of prehospital RA, particularly in paramedic-based EMS systems. FICB may represent a suitable option, as it is widely described as a simple and safe technique with a low complication rate [41]. The block targets a fascial plane rather than a specific nerve and can be performed using landmark-based approaches. The injection site is away from major blood vessels and nerves, reducing the risk of accidental intravascular injection [41, 42]. These features may explain why FICB was among the techniques most frequently supported by respondents and why it is increasingly considered for emergency and prehospital analgesia, particularly given the previously discussed concern regarding LAST and the limited availability of lipid therapy in many prehospital systems.

Beyond safety, respondents expressed concerns about potential delays due to procedural time. Nevertheless, current evidence indicates that prehospital PNBs, when performed by trained providers, do not cause clinically significant delays to definitive treatment (e.g., surgical intervention) and do not compromise overall patient outcomes. In particular, for cases of isolated femoral fractures with stable vital signs, the minimal delay is unlikely to have clinical relevance and may even reduce the need for additional analgesia later in the clinical course [43]. While FNBs take longer to administer than systemic analgesia, they do not significantly impact patient care [3, 44, 45]. FICBs require more time than other interventions, but total on-scene time remains comparable to systemic treatments [26, 39]. These findings suggest that although PNBs may slightly extend procedural time, the impact on patient care remains negligible. Large-scale studies are needed to confirm feasibility.

Concerns about infection were also mentioned, though evidence suggests that the risk is minimal for single-injection ultrasound-guided PNBs. Most hip fracture falls occur in clean, dry home environments. Current guidelines recommend sterile gloves, sterile single-use gel, and sterile probe covers for ultrasound-guided PNBs, with additional precautions primarily reserved for catheter-based techniques [46]. Because most ambulances are equipped for sterile invasive procedures, adherence to these hygiene standards appears feasible in prehospital settings.

Interestingly, respondents placed little emphasis on obtaining explicit informed consent for PNBs in emergencies, suggesting general acceptance of RA in urgent situations.

This aligns with other emergency procedures where implied consent is typically assumed when interventions are in the patient’s best interest [47]. 

### Strengths and limitations of this study

The majority of respondents reported extensive clinical experience and stated that they perform RA independently, without supervision or assistance, as part of their routine clinical practice in hospital settings. Most participants reported working in larger hospitals, including academic and regional centers, suggesting a potentially broad exposure to clinical workflows and safety considerations relevant to the use of PNBs. Their qualified perspectives therefore offer valuable insights into the feasibility and prerequisites for implementing such techniques in prehospital care. By capturing these expert views, the study highlights key opportunities, challenges, and barriers to extending PNB beyond the hospital environment.

Nevertheless, our study has several limitations.

First, distributing the survey exclusively through the ESRA newsletter likely introduced a selection bias toward individuals with a preexisting interest in the topic. As a result, clinicians from other specialties involved in emergency care, as well as anesthesiologists not affiliated with ESRA, were not reached. While this targeted approach ensured expert-level responses, it also limits the generalizability of our findings beyond the anesthesiology community. This recruitment strategy may therefore introduce selection bias toward clinicians with a particular interest in RA. Future studies should also include other professional groups involved in prehospital emergency care to ensure a broader and more representative perspective.

Second, the low overall response rate constitutes a limitation and may contribute to non-response bias, as only a small proportion of ESRA members participated. One potential reason why many did not click on the survey link may be that they were not actively involved in prehospital care and therefore did not feel addressed by the study. Although the response rate relative to the entire mailing list appears low (3%), such rates are common in large-scale online surveys targeting physicians [48]. However, the high completion rate among those who accessed the survey suggests genuine engagement, and a substantial number of fully completed questionnaires were available for analysis. Nevertheless, the findings may not be fully representative of the broader ESRA membership or other clinicians involved in prehospital emergency care and should therefore be interpreted with appropriate caution.

Third, while paramedics represent the primary providers of prehospital emergency care in many European EMS systems, they were not included in this study, which was focused on physician respondents. As a result, the perspectives of non-physician EMS providers may not be fully represented.

Fourth, the survey did not collect detailed information on respondents’ level of experience in EMS activities. Therefore, the degree of direct prehospital experience among participants may have varied within the study population, and some respondents may have had limited or no direct EMS experience, which may further limit the generalizability of the findings.

Finally, despite the use of browser cookies, the possibility of multiple submissions by the same individual cannot be entirely excluded.

Taken together, these factors may limit the external validity of the findings, and the results should therefore be interpreted primarily as reflecting expert perspectives rather than representing all EMS professionals.

## Conclusion

While RA is a core competency within anesthesiology, its application in prehospital emergency medicine remains limited. Most respondents supported the use of prehospital RA, yet only a minority reported its current implementation within their local EMS systems. FICB and FNB were identified as the most feasible options for analgesia in proximal femur fractures, whereas fewer respondents considered PNBs appropriate for isolated shoulder dislocations or rib fractures. Ultrasound was regarded as the most essential prerequisite, although its availability remains inconsistent across prehospital services. Respondents emphasized the importance of structured physician training and standardized protocols, while more than half opposed the performance of PNBs by non-physician personnel. Principal concerns included limited provider experience, procedural duration, and complications such as LAST. Notably, explicit informed consent was considered less critical in emergency scenarios.

These findings provide expert perspectives and suggest that prehospital RA may be feasible and clinically meaningful when implemented within a structured, safety-oriented framework. However, successful implementation may vary depending on local EMS structures, particularly between physician-staffed and paramedic-based systems. Future prospective implementation studies are required to evaluate the feasibility, safety, clinical effectiveness, and cost-effectiveness of prehospital PNBs across different EMS systems. 

## Data Availability

Data are available upon reasonable request. All data relevant to the study are included in the article or uploaded as online supplemental information.

## Declarations

### Clinical trial number

The study was prospectively registered in the German Clinical Trials Register (DRKS; registration number DRKS00034436) on 24 June 2024.

## Abbreviations

ECG: Electrocardiography
ED: Emergency department
EMS: Emergency medical services
ESPB: Erector spinae plane block
ESRA: The European Society of Regional Anaesthesia and Pain Therapy
FICB: Fascia iliaca compartment block
FNB: Femoral nerve block
GA: General anesthesia
ISB: Interscalene brachial plexus block
LAST: Local anesthetic systemic toxicity
NIBP: Non-invasive blood pressure measurement
PECS: Interpectoral and pecto-serratus plane block
PENG: Pericapsular nerve group block
PNB: Peripheral nerve block
PVB: Paravertebral block
RA: Regional anesthesia
SOP: Standard operating procedures
